# Assessing frailty in hemodialysis patients: utility of a self-rated visual analogue scale and the role of routine parameters in clinical prediction

**DOI:** 10.1186/s12882-026-05218-z

**Published:** 2026-07-22

**Authors:** Lena Schulte-Kemna, M. Künzig, C. Hertneck, M. Denkinger, R. van Erp, W. Koch, D. Dallmeier, M. Kächele, L. Bettac, B. Schröppel

**Affiliations:** 1 Division of Nephrology, University Hospital Ulm, Ulm, Germany; 2 University of Ulm, Ulm, Germany; 3 Institute for Geriatric Research, University of Ulm, Ulm, Germany; 4 Agaplesion Bethesda Hospital, Ulm, Germany; 5 B.Braun Gesundheitszentrum Neu-Ulm MVZ GmbH, Neu-Ulm, Germany; 6 School of Public Health, Boston University, Boston, USA

**Keywords:** Frailty, Hemodialysis, Functional status, Self-assessment, Geriatric assessment

## Abstract

**Background:**

There is still no established consensus on the optimal frailty screening tool for routine clinical application in patients with chronic kidney disease (CKD). We evaluated the accuracy of a visual analogue scale (VAS) for frailty screening and identified clinical predictors of frailty based on the modified Fried criteria.

**Methods:**

This cross-sectional, multicenter study included 122 adult patients on hemodialysis. Frailty status was assessed using modified Fried criteria and a 5-point self-rated VAS. The agreement between these two methods was evaluated using Cohen’s Kappa. For the frailty prediction model we applied logistic regression with backwards selection among a set of clinical parameters associated with this condition. Pre-frail and robust individuals were analyzed as non-frail.

**Results:**

Median age was 68 years, 71% were male and 42.6% were considered frail according to Fried criteria. Cohen’s Kappa was 0.36 (95% CI [0.21, 0.51]), indicating a fair level of agreement between VAS and Fried criteria. The VAS correctly identified most non-frail patients (specificity 94.3%, PPV 82.6%) while it missed a significant number of frail patients (sensitivity 36.5%, NPV 67.0%). Patients at risk for frailty based on the modified Fried criteria were more likely to have a central venous hemodialysis catheter, lower diuresis, lower creatinine and albumin, a higher prevalence of cardiovascular disease and use of analgesics. A model including these factors demonstrated good predictive performance with an AUC of 0.816 [95% CI 0.74, 0.90].

**Conclusion:**

A self-rated VAS for frailty has the potential to simplify frailty screening and enhance its integration into routine nephrology care. A model based on routine parameters can support the identification of frail patients.

**Supplementary Information:**

The online version contains supplementary material available at 10.1186/s12882-026-05218-z.

## Key message

• Self-assessment of frailty status using a visual analogue scale (VAS) has the potential to become an acceptable and convenient tool for frailty screening in people undergoing hemodialysis. Adjustments might improve its discriminatory properties before broad clinical application is possible.

• A model using routine clinical and laboratory information (dialysis access, presence of CVD, residual diuresis, regular analgesic medication, creatinine and albumin) shows good predictive performance and can help identify frail patients on hemodialysis. Extracted from digital health records, this could support frailty risk-assessment.

**Supplementary Information:**

The online version contains supplementary material available at 10.1186/s12882-026-05218-z.

## Introduction

Frailty describes a complex syndrome of increased vulnerability to stress factors as a consequence of age-related degeneration in various organ systems [1]. Frailty occurs earlier and its prevalence is higher in patients with chronic kidney disease (CKD) compared to the general population [2]. In patients with CKD frailty is associated with a higher mortality rate, more frequent hospitalizations, an increased risk of falls and fractures, reduced quality of life (QoL), cognitive and functional decline and decreased access to kidney transplantation [3–7]. The identification of frailty allows for detection of modifiable risk factors and initiation of targeted interventions. Giving important prognostic information – superior to many biochemical tests, age or comorbidity – frailty status can also guide clinical decision making and prompt discussion about advance-care planning [8]. The European Renal Best Practice (ERBP) Guideline on the management of older patients with CKD recommends an assessment of functional status and possibly frailty status on a regular basis with the intention of identifying those who would benefit from a more in-depth geriatric assessment and rehabilitation program [9]. However, frailty screening has not become part of routine care. A lack of consensus on a screening tool and screening intervals, the time-consuming nature of many tests and insufficient training in frailty assessment are possible reasons.

The primary objective of this study was to evaluate frailty self-assessment based on a 5-point visual-analogue scale (VAS) compared to modified Fried criteria. As a secondary objective, we identified clinical predictors for frailty based on the modified Fried criteria. Both strategies could be implemented easily in clinical practice and simplify frailty screening in hemodialysis patients.

## Materials and methods

### Study population

This cross-sectional, multicenter study included 122 adult patients who received chronic hemodialysis in three dialysis centers between April and June 2021. Of the 131 patients initially approached for this study 9 (6.87%) were excluded: 3 declined to participate, 5 met predefined exclusion criteria (age < 18 years, estimated survival < 3 months and insufficient German language skills) and one patient was on intermittent peritoneal dialysis. All participants provided written informed consent to participate in the study. The ethics committee of the University of Ulm approved the study (application no. 47/20).

Demographic variables, current medication including erythropoiesis-stimulating agents (ESA), iron supplements, antidepressants and analgesics, pre-defined conditions like cardiovascular disease (CVD), heart failure (HF), stroke, diabetes, malignancy, chronic obstructive pulmonary disease (COPD), peripheral artery disease (PAD), type of dialysis access, dialysis vintage and hospitalizations were obtained from the patient chart. Laboratory values were collected from the monthly routine evaluation closest to the assessment and included serum creatinine, c-reactive protein (CRP), serum-albumin, hemoglobin, ferritin, transferrin-saturation (TSAT), phosphate and bicarbonate. The included parameters were chosen based on established or potential associations with frailty status.

### Frailty

Frailty status was defined based on the Fried Model [1], with four of the five criteria modified as follows: (1) Self-reported exhaustion (exhaustion in the last 4 weeks rated as “most of the time” or “all of the time”) using a single-item from the validated German language version of the Medical Outcome Short Form-36 (SF-36) [10]. (2) Weakness (grip strength < 33 kg in male, and < 21 kg in female) according to data from the representative German population-based cohort study Activity and Function in the Elderly in Ulm (ActiFE) [11]. Both hands were tested and the highest value used for data analysis. (3) Slow walking speed (≥ 15 s according to the Timed-up-and-go (TUG) Test using a 3 m distance, one trial was performed) [12, 13]. (4) Low physical activity level according to the “German Health Interview and Examination Survey for Adults (DEGS1)” (self-reported no physical activity: “On how many days a week are you physically active in a way that you start to sweat or get out of breath? An average week is meant ?” and “how often do you participate in sports in an average week?” is answered with “zero days” and “no sports” respectively) [14]. Unintentional weight loss of 5 kg or more matched the original Fried criteria [1]. Patients meeting 3 of the 5 criteria were categorized as frail. Performance tests and interviews were carried out by the same person in the same standardized manner across all centers. Interview and self-assessment were done during the hemodialysis session. Performance based tests were accomplished the same day after the dialysis session.

### 5-point visual-analogue scale (VAS)

**Fig. 1:**
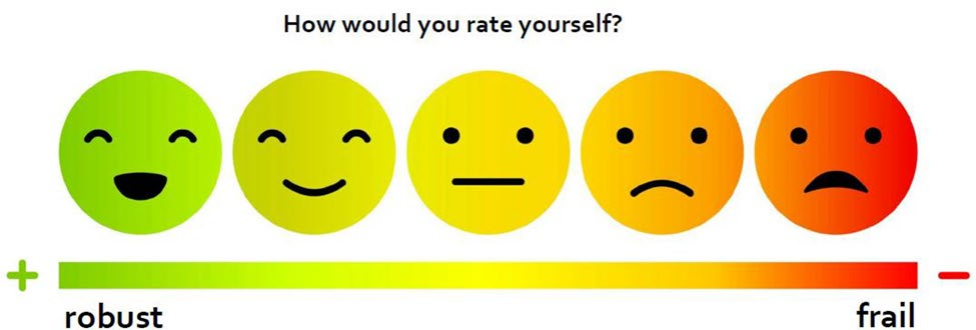
Frailty self-assessment using a Visual Analogue Scale (VAS)

Patients received a definition for frailty as follows: *“Frailty describes a state of reduced resilience and resistance to stress factors such as illness*,* operations*,* hospital stays and falls. If someone is frail he or she lacks the physical reserves to recover from these stress factors”* (translated from German). Based on this information patients were requested to self-assess their frailty status on a 5-point VAS showing 5 different smiley faces (Fig. 1). Patients were considered frail selecting smiley number 4 and 5, pre-frail selecting smiley number 2 and 3 and robust selecting smiley number 1. Self-assessment took on average 5 min to complete.

### Statistical analysis

For data analysis, patients categorized as pre-frail or robust were grouped as “non-frail.” A descriptive analysis was performed to compare frail and non-frail patients based on Fried’s criteria. Additionally, both groups were compared inferentially, depending on the structure of the variables. Categorical variables were analyzed using the Chi-square test, only if cell frequency was less than 6 Fisher’s exact test was applied. Continuous variables with a normal distribution were compared using an independent samples t-test, while non-normally distributed variables were analyzed using the Mann-Whitney U test. Sensitivity, specificity, positive predictive value (PPV), and negative predictive value (NPV) were calculated to evaluate the diagnostic performance of the VAS compared to the Fried criteria as the gold standard. Cohen’s Kappa was calculated to assess the level of agreement between the two measures. Additionally, agreement status was further analyzed for differences in demographic variables, using the same tests as for frailty status, except for continuous variables: parametric variables were analyzed with one-way ANOVA, and non-parametric variables with the Kolmogorov-Smirnov test. In a secondary analysis we evaluated the discriminatory properties of the VAS with a cutoff for frailty when patients selected smiley 3,4 or 5.

**Fig. 2:**
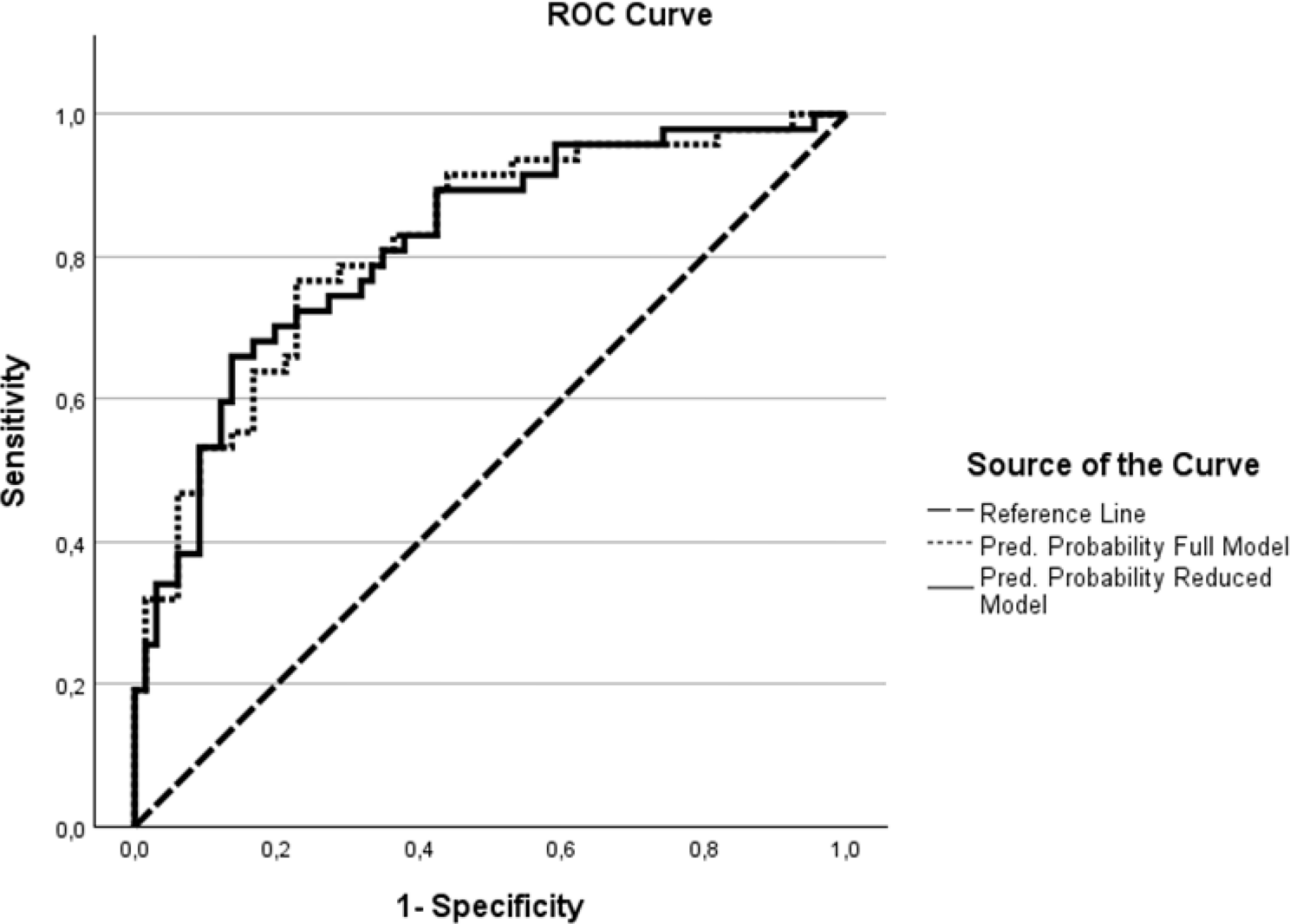
ROC Frailty in backward selection

To identify clinical predictors for frailty a logistic regression with backward selection was performed, including following potential predictors with a p-value ≤ 0.1 from the inferential analysis: age, CVC, diuresis (ml/24 h), albumin, creatinine, CVD, hospitalizations in the previous 12 months, analgesic use. Before proceeding, multicollinearity among predictors was assessed using the variance inflation factor (VIF) with a threshold < 5. Backward elimination was performed using the likelihood ratio statistic, comparing the current model to versions in which one predictor was removed at a time. Predictors were excluded when the likelihood-ratio test indicated that their removal did not significantly worsen model fit (*p* > 0.10; see Supplement Table 1). Following model selection, predicted probabilities were calculated, and a receiver operating characteristic (ROC) curve was constructed to assess model performance (Fig. 2). The area under the curve (AUC) was used to evaluate the model’s discriminatory ability. All calculations were performed using SPSS Version 29.0.1.0.

## Results

The median age of the included 122 patients was 68 years, 71% were male. Table 1 shows the descriptive characteristics for the study population as well as stratified by frailty status. Overall, 52 (42.6%) patients were categorized as frail according to the Fried criteria. Frail patients had significantly less frequently an arterio-venous fistula/graft (AVF/AVG), were significantly older in age, had a significantly lower residual diuresis as well as lower creatinine and albumin values. In addition, frailty was associated with a higher prevalence of stroke, PAD, history of hospitalization and use of analgesics.

**Table 1:** Demographics at the time of data collection. Frailty-status according to Fried

	*n* = 122	non-frail *n* = 70	*N*/Missing	Frail *n* = 52	*N*/Missing	*p*-value
Male, *n* (%)	86 (70.5)	52 (74.3)	70/0	34 (65.4)	52/0	0.286
Age (y), *MD* (Q1; Q3)	68 (56.8; 78.3)	66.0 (53.5, 75.3)	70/0	71.5 (59.8,81.0)	52/0	**0.040**
Arterio-venous fistula/graft, n (%)	89 (73.0)	60 (85.7)	70/0	29 (55.8)	52/0	**< 0.001**
BMI (kg/m2), *MD* (Q1; Q3)	26.5 (23.8; 30.6)	27.2 (24.0, 30.9)	70/0	26.2 (23.0, 30.1)	51/1	0.537
Diuresis (ml/24 h), MD (Q1; Q3)	600.0 (100.0; 1700.0)	1000.0 (177.5, 1937.5)	68/2	300.0 (0.0, 1275.0)	48/4	**0.006**
Creatinine (mg/dl), *MD* (Q1; Q3)	7.4 (6.2, 9.9)	8.3 (6.5, 10.8)	70/0	7.0 (5.8, 8.5)	52/0	**0.019**
Albumin (g/l), *MD* (Q1; Q3)	36.5 (33.1, 39.8)	37.1 (34.5, 40.0)	69/1	35.4 (30.7, 39.0)	51/1	0.037
Phosphate (mmol/l), M ± SD	2.0 ± 0.6	2.1 ± 0.7	70/0	2.0 ± 0.6	51/1	0.228
Hb (g/dl), M ± SD	11.0 ± 1.3	11.1 ± 1.2	69/1	10.8 ± 1.3	52/0	0.217
Ferritin (µg/l), *MD* (Q1; Q3)	568.2 (350.3, 1030.4)	536.8 (314.0, 998.9)	70/0	732.0 (414.6 1156.6)	50/2	0.338
Transferrin Saturation (%), *MD* (Q1; Q3)	23.0 (17.0, 28.5)	22.6 (17.2, 25.4)	70/0	24.6 (16.0, 31.8)	52/0	0.230
CRP (mg/l), MD (Q1; Q3)	6.3 (3.3, 13.9)	5.8 (2.8, 11.5)	70/0	7.7 (3.6, 21.1)	51/1	0.154
Diabetes, n (%)	38 (31.1)	19 (27.1)	70/0	19 (36.5)	52/0	0.268
CAD, n (%)	35 (28.7)	20 (28.6)	70/0	15 (28.8)	52/0	0.974
Heart Failure, n (%)	15 (12.3)	5 (8.6)	70/0	10 (19.2)	52/0	0.054
Hypertension, n (%)	107 (87.7)	58 (82.9)	70/0	49 (94.2)	52/0	0.059
PAD, n (%)	9 (7.4)	2 (2.9)	70/0	7 (13.5)	52/0	**0.036**
Stroke, n (%)	7 (5.7)	1 (1.4)	70/0	6 (11.5)	52/0	**0.041**
COPD, n (%)	5 (4.1)	3 (4.2)	70/0	2 (3.8)	52/0	1.000
Cancer, n (%)	19 (15.6)	9 (12.9)	70/0	10 (19.2)	52/0	0.337
Hospitalisation/12 months, n (%)	62 (50.8)	29 (41.4)	70/0	33 (63.5)	52/0	**0.016**
ESA n (%)	103 (85.1)	58 (84.1)	69/1	45 (86.5)	52/0	0.704
Intravenous Iron n (%)	104 (86.0)	62 (89.9)	69/1	42 (80.8)	52/0	0.155
Antidepressants n (%)	9 (7.4)	4 (5.7)	69/1	5 (9.6)	52/0	0.496
Analgesics n (%)	54 (44.6)	24 (34.8)	69/1	30 (57.7)	52/0	**0.012**

BMI: body-mass-index; Hb: Hemoglobin; CRP: c-reactive protein; UACR: urinary albumin-creatinine-ratio; Crea: Creatinine; CAD: coronary artery disease; PAD: peripheral artery disease; COPD: chronic obstructive pulmonary disease; ESA: Erythropoiesis stimulating agents

### Accuracy of VAS as frailty screening tool

Descriptively, there are fewer frail people identified by VAS (*n* = 23) than by Fried (*n* = 52). Supplementary Fig. 1 shows the distribution of the VAS Score. The sensitivity of VAS was 36.5% while the specificity laid in 94.3%. The PPV was 82.6% and the NPV was 67.0%. Cohen’s Kappa was 0.36 (95% CI [0.21, 0.51]), indicating a fair level of agreement between VAS and Fried (Table 2). Table 3 shows participant characteristics according to the agreement status. Participants who misjudged their frailty status (*n* = 37, 30.3%) had a significantly lower residual diuresis, took analgesic medications more often and were more frequently diagnosed with HF than participants who correctly assessed their frailty status. Using an AVF/AVG as dialysis access was associated with the correct identification as non-frail while hospitalization within the last 12 months and presence of PAD was associated with the correct identification as frail.

**Table 2:** Self-assessment of frailty status by VAS compared to Fried

	non-frail by Fried	frail by Fried	total
VAS non-frail, *n*	66	33	99
VAS frail, n	4	19	23
total	70	52	122

VAS, visual analogue scale

**Table 3:**
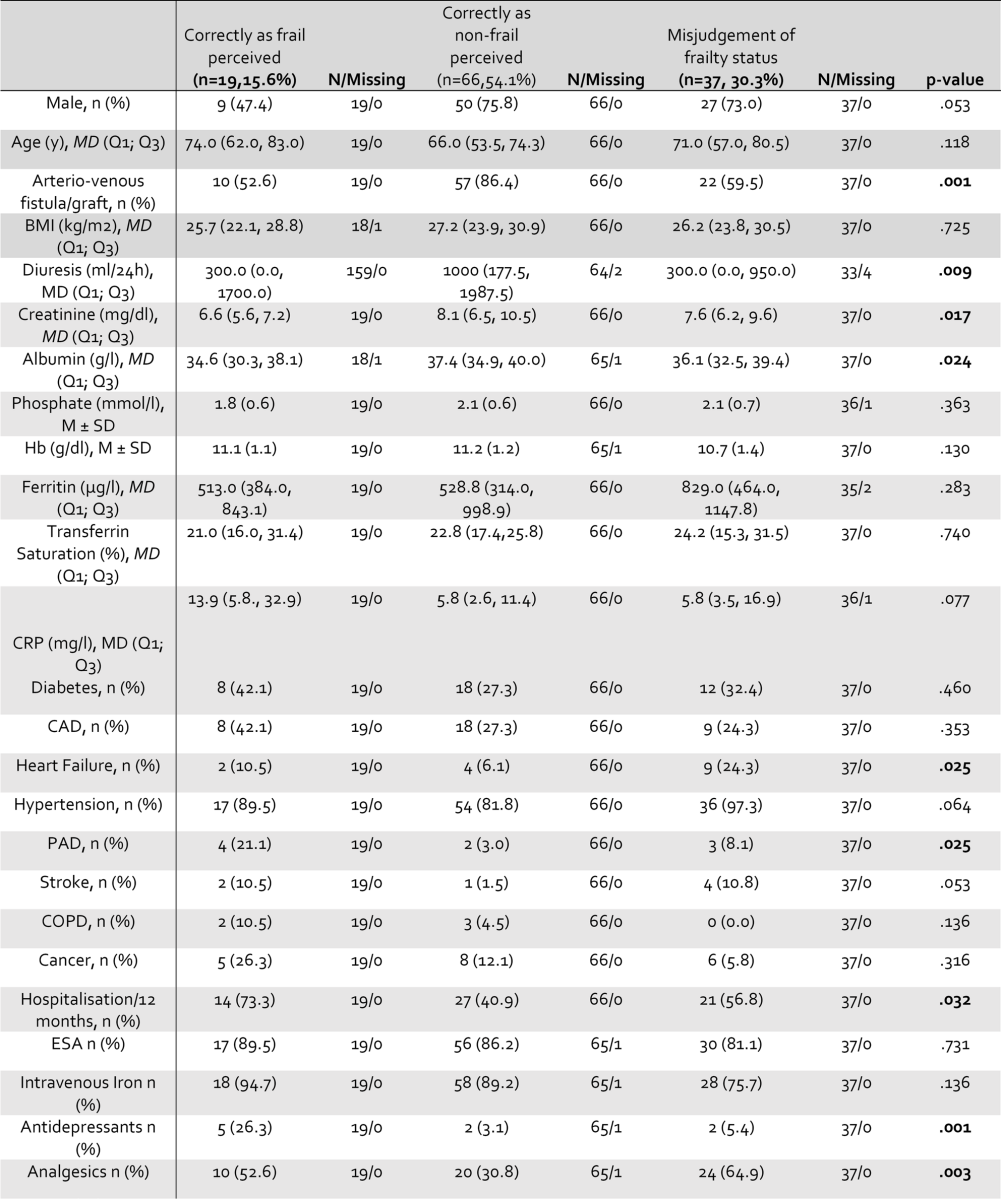
Demographics stratified by frailty agreement

BMI: body-mass-index; Hb: Hemoglobin; CRP: c-reactive protein; UACR: urinary albumin-creatinine-ratio; Crea: Creatinine; CAD: coronary artery disease; PAD: peripheral artery disease; COPD: chronic obstructive pulmonary disease; ESA: Erythropoiesis stimulating agent

The secondary analysis showed that lowering the cutoff for frailty on the VAS to smiley number 3 leads to a specificity of 52.9% and a sensitivity of 94.2% (Supplementary Table 3). Accordingly, the PPV was 59.8% and the NPV 92.5%.

### Clinical predictors of frailty

The initial (full) model included eight predictors. Through iterative removal of non-significant variables as described in methods, age and hospitalization were excluded after three elimination steps, resulting in the final (reduced) model. This included central venous catheter (CVC) as dialysis access, diuresis, albumin, creatinine, CVD and the use of analgesics (Table 4; Fig. 2). Among predictors, CVC and CVD were statistically significant associated with an increased odd for frailty with an OR 5.2 (95% CI [1.7, 15.7]) and 5.2 (95% CI [1.01, 26.9]), respectively. However, the wide confidence intervals indicate limited precision and may be the result of a small sample size. On the other hand, higher creatinine levels were statistically significant associated with a decreased likelihood for frailty (OR 0.8, 95% CI [0.7, 0.95]).

**Table 4:** Backward selection

	Full model		Reduced model	
	OR (95% KI)	*P*-Value	OR (95% KI)	*P*-Value
Age	1.0 [1.0,1.1]	0.246		
Central venous catheter	4.4 [1.4, 13.5]	0.011	5.2 [1.7, 15.7]	0.004
Diuresis (ml/24 h	0.99 [0.99,1.00]^¥^	0.011	0.99 [0.99,1.00]	0.011
Albumin (g/l	0.9 [0.8, 1.0] ^¥^	0.138	0.9 [0.8,1.0]	0.108
Creatinine (mg/dl)	0.9 [0.7, 1.0] ^¥^	0.133	0.8 [0.7, 0.95]	0.014
Cardiovascular disease	5.1 [1.0,26.2]	0.049	5.2 [1.01, 26.9]	0.048
Hospitalisation /12 months	0.6 [0.2,1.4]	0.216		
Analgesics	2.1 [0–8,5 − 3]	0.113	2.1[0.9, 5.3]	0.102
Constant		0.652		0.201

Regarding model quality, the pseudo R² values indicate a moderate proportion of explained variance (Cox & Snell R² = 0.285, Nagelkerke R² = 0.375). The AUC demonstrates good predictive performance, with the full model achieving an AUC of 0.818 (95% CI [0.739, 0.897]) and the reduced model showing a comparable AUC of 0.816 (95% CI [0.737, 0.896]). The Hosmer-Lemeshow goodness-of-fit test was non-significant (χ²(8) = 5.7, *p* = 0.683), suggesting an adequate fit of the model to the data. Additionally, the likelihood ratio difference tests showed no significant loss in fit when moving from the full model to the second model (χ²(1) = 1.27, *p* = 0.241) and from the second to the final reduced model (χ²(1) = 1.1, *p* = 0.299), supporting the use of the more parsimonious reduced model.

## Discussion

In this cross-sectional, multicenter study we evaluated the accuracy of a 5-point VAS as a self-assessment tool for frailty in patients on chronic hemodialysis. Our findings demonstrate fair agreement between the VAS self-assessment and the Fried criteria. Although the sensitivity for the detection of frailty was low, the accuracy for the detection of non-frail patients was high. In addition, we found that a model based on routine clinical parameters – including cardiovascular disease status, dialysis access type, albumin and creatinine levels, residual urine output, and analgesic use – provided good predictive performance for identifying frailty.

Our sample had a frailty prevalence of 42.6% according to the modified Fried criteria, which is in agreement with other studies reporting a prevalence of frailty between 24% and 73% in the dialysis population [15].

Frailty self-assessment using the 5-point VAS was performed during the dialysis session. Although Cohen’s kappa indicated fair agreement between the VAS and the Fried phenotype, the VAS showed high specificity (95%) for identifying non-frail patients but failed to identify a substantial proportion of frail patients, resulting in low sensitivity. Taken together, these findings suggest that the VAS, as implemented here, may be useful for ruling out frailty in dialysis patients. However, its limited sensitivity for identifying frail individuals may restrict its application as a frailty screening tool. A lower cutoff for frailty in the secondary analysis resulted in a higher sensitivity and a lower specificity, increasing the number of false positives. As positive frailty screenings require comprehensive assessments for confirmation, efforts to increase sensitivity must consider the corresponding increase in resources devoted to recognizing false positives.

Our results suggest that the most robust and the most frail patients show better judgement in their frailty status.

Self-assessment of frailty status has been tested before. Salter et al. evaluated the relationship between perceived (by nephrologist, nurse practitioner and dialysis patient) and measured (Fried criteria) frailty and found poor agreement in all groups [16]. No information was given about the concept of frailty, as we did our study. However, we did not assess patients’ understanding of the information provided.

We believe that further exploration of frailty self-assessment in the dialysis population is important. First, different tools have been evaluated in the CKD population with most robust evidence for the Fried phenotype [17]. However, performance-based tests are difficult to realize in the dialysis setting as performance measures might be influenced by pre-dialysis volume overload or post-dialysis fatigue and hypotension. A lack of space for walking assessment and time constraints due to transportation and dialysis schedules can further complicate its application. Second, self-assessment with VAS is widely used in clinical practice as a simple and intuitive approach to measure patient reported outcomes such as pain, itch, fatigue or quality of life [18, 19]. Third, self-rated health or performance status is an important prognostic marker of its own and showed strong associations with mortality in dialysis patients [20]. In a cohort of older cancer patients, a single-item self-rated health question identified frailty and geriatric impairments with high sensitivity and specificity [21].

The main strengths of the VAS are its simplicity and convenience. Its value for the identification of frail patients should be further examined. Since pre-frail patients might benefit more from targeted interventions than frail patients, further discrimination between non-frail, pre-frail and frail needs to be assessed [22]. However, we recognize that understanding the concept of frailty may be challenging for patients and that our explanation of frailty may have been too abstract. A more accessible description of frailty and the use of pictographs similar to those in the Clinical Frailty Scale [23] rather than smileys, may improve the sensitivity of the VAS. This highlights the need for a consensus-based frailty definition that is accessible to patients and their family members.

As a secondary objective we identified predictors for frailty (according to modified Fried criteria) among routine clinical parameters. Several variables were strongly associated with frailty. A model incorporating residual diuresis, dialysis access type, presence of CVD, analgesic use, creatinine and albumin levels showed good predictive performance for frailty. This information could support the identification of patients with frailty. Furthermore, extraction of this data from digital health records enables automation of risk assessment. Whether a combination of this prediction model with (self-)assessment enhances or simplifies frailty screening in people on hemodialysis deserves further exploration.

As previously described we found a significant correlation between the use of a CVC as dialysis access and frailty [24]. A prospective cohort study of 40 patients undergoing new AVF or AVG placement demonstrated a significantly higher AVF maturation failure in frail compared to non-frail patients [25]. Even in patients with a functioning AVF or AVG, frailty is associated with a more than 2-fold higher risk of vascular access thrombosis [26]. Endothelial dysfunction, chronic inflammation and increased oxidative stress may represent possible overlapping mechanisms both in the pathogenesis of frailty and vascular access dysfunction [27]. We did not collect data on vascular access failure; therefore, it remains unclear whether the higher prevalence of CVC use among frail patients reflects previous vascular access failure or serves as a surrogate marker of comorbidity influencing dialysis access selection. These results, however, support the current KDOQI Clinical Practice Guidelines for Vascular Access, which advocate an individualized “Life-Plan” approach considering patients’ preferences, medical situation, life goals, and functional status [28]. The incorporation of frailty in dialysis access planning could provide important information about the most suitable type of vascular access, though more studies are needed to confirm this approach.

In addition, a higher creatinine value was associated with a decreased risk of frailty. Since creatinine is rather a marker of muscle mass than of kidney function in people with ESRD this is not surprising. The relationship between creatinine, muscle strength and physical function has been described before [29]. The association between analgesic use and frailty is possibly the result of a higher pain-burden in frail individuals [30, 31]. Chronic pain potentially contributes to the development of frailty since it is correlated with depression, sleep-disturbances, limited mobility and an increased risk of falls [32–34]. Polypharmacy and the use of potentially inappropriate medications (PIM) on the other hand are also associated with frailty [35, 36]. Periodic medication review and frailty screening in people with polypharmacy is therefore advisable.

Frailty screening is only the first step and (self-)assessment as frail should trigger in-depth investigations in potentially reversible factors and initiation of targeted interventions [37, 38]. The gold standard to assess and manage frailty in older people is a comprehensive geriatric assessment (CGA), a multidimensional and interdisciplinary diagnostic process evaluating mobility, cognition, nutrition, psychological and social situation [39]. Its positive effects on both functionality and survival have been confirmed in various settings [40]. Implementation of a CGA in people with CKD demonstrated value in guiding CKD-care and dialysis decision-making, although studies on the impact on frailty status and mortality are lacking [41]. The GOAL-Study evaluated important health outcomes of a CGA in frail older people with CKD; results have not been published yet [42]. Outside the context of a CGA, exercise-based interventions have demonstrated potential to stabilize or improve frailty parameters among individuals with CKD [43, 44]. Successful frailty interventions will likely have to address multiple contributing factors. A combination of exercise, nutritional and psychological counseling in people starting hemodialysis has shown to stabilize and prevent further deterioration of health and performance status in the pre-frail and frail [45].

Simplification of frailty screening in the hemodialysis population will result in an increased identification of frail patients. Implementation of a CGA and initiation of targeted interventions in all these patients requires human and financial resources. Given the increasing shortage of skilled healthcare professionals and cost pressure in the healthcare sector, health economic evaluations alongside patient-reported and clinical outcomes are necessary to promote essential geriatric and person-centered care for people with CKD.

## Limitations

Our study has several limitations: (1) While the frailty definition provided to the patients was drawn from the literature, it was developed without patient involvement and patient understanding was not tested. (2) The frailty definition based on the Fried criteria included modifications to some of the original variables. (3) We did not collect data on cognitive impairment, nor did we perform a cognitive assessment. Misclassification in the self-assessment resulting from cognitive impairment cannot be excluded. (4) Self-assessment of frailty during dialysis sessions appears to be feasible in real-world clinical practice. However, the optimal timing for frailty self-assessment in relation to dialysis treatment remains unclear. (5) We did not capture dialysis-related stress-parameters like interdialytic weight gain, ultrafiltration volume or intradialytic hypotension – which could have influenced frailty assessment. (6) Performance-based Fried criteria were assessed after the dialysis session. While a detrimental effect on grip strength post-dialysis has been reported, the impact of dialysis on walking speed measured pre- and post-dialysis remains controversial [46–48]. Therefore, assessment of frailty according to the Fried criteria may be influenced by post-dialysis fatigue and hypotension. (7) Self-assessment of frailty was done at a single timepoint without follow-up of frailty-status or outcome. Future studies are necessary to validate our findings and the association of frailty self-assessment using a VAS with outcomes, especially in the context of interventions.

## Conclusion

Taken together, self-assessment of frailty status using a VAS in dialysis patients can be helpful in ruling out frailty and identifying individuals who might benefit from a more comprehensive assessment. It has therefore the potential to simplify frailty screening, improve the efficient use of limited resources and enhance its integration into routine nephrology care. Recognition of frailty should trigger further investigations, ideally a CGA, to identify potentially modifiable risk factors and enable a multidimensional and interdisciplinary approach to address frailty and to develop individualized patient-centered treatment strategies.

## Supplementary Information

Below is the link to the electronic supplementary material.

Supplementary Material 1

## Data Availability

The datasets used and/or analysed during the current study are available from the corresponding author on reasonable request.

## Abbreviations

AVF: Arterio-venous fistula
AVG: Arterio-venous graft
AUC: Area under the curve
CGA: Comprehensive geriatric assessment
CKD: Chronic kidney disease
COPD: Chronic obstructive pulmonary disease
CRP: C-reactive protein
CVC: Central venous catheter
CVD: Cardiovascular disease
ESA: Erythropoiesis-stimulating agents
HF: Heart failure
NPV: Negative predictive value
PAD: Peripheral artery disease
PPV: Positive predictive value
QoL: Quality of life
ROC: Receiver operating characteristic
TSAT: Transferrin saturation
VAS: Visual analogue scale
VIF: Variance inflation factor
