# Supplementary material for: Assessing frailty in hemodialysis patients: utility of a self-rated visual analogue scale and the role of routine parameters in clinical prediction
Source: BMC Nephrol. 2026 Jul 22;27:443. doi: 10.1186/s12882-026-05218-z (PMC13397956; doi:10.1186/s12882-026-05218-z)

**Supplement Table 1: Modeling of removed terms**

|  | **Variable** | **Model Log-Likelihood** | **Change in -2 Log-Likelihood** | **Degrees of freedom (df)** | **Significance of change** |
| --- | --- | --- | --- | --- | --- |
| **Step 1** | Age | -57,748 | 1,372 | 1 | ,241 |
|  | Central venous catheter | -60,568 | 7,011 | 1 | ,008 |
|  | Diuresis (ml/24h | -60,714 | 7,305 | 1 | ,007 |
|  | Albumin (g/l | -58,325 | 2,525 | 1 | ,112 |
|  | Creatinine (mg/dl) | -58,229 | 2,333 | 1 | ,127 |
|  | Cardiovascular disease | -59,322 | 4,520 | 1 | ,033 |
|  | Hospitalisation /12 months | -57,828 | 1,533 | 1 | ,216 |
|  | Analgesics | -58,337 | 2,550 | 1 | ,110 |
| **Step 2** | Central venous catheter | -61,808 | 8,119 | 1 | ,004 |
|  | Diuresis (ml/24h | -61,144 | 6,792 | 1 | ,009 |
|  | Albumin (g/l | -59,064 | 2,632 | 1 | ,105 |
|  | Creatinine (mg/dl) | -60,412 | 5,328 | 1 | ,021 |
|  | Cardiovascular disease | -59,898 | 4,299 | 1 | ,038 |
|  | Hospitalisation /12 months | -58,288 | 1,080 | 1 | ,299 |
|  | Analgesics | -59,145 | 2,794 | 1 | ,095 |
| **Step 3** | Central venous catheter | -62,887 | 9,198 | 1 | ,002 |
|  | Diuresis (ml/24h | -61,946 | 7,315 | 1 | ,007 |
|  | Albumin (g/l | -59,765 | 2,954 | 1 | ,086 |
|  | Creatinine (mg/dl) | -61,550 | 6,524 | 1 | ,011 |
|  | Cardiovascular disease | -60,548 | 4,519 | 1 | ,034 |
|  | Analgesics | -59,648 | 2,720 | 1 | ,099 |

**Supplement Table 2: Variance Inflation Factor**

| Model | | Collinearity Statistics | |
| --- | --- | --- | --- |
|  |  | Tolerance | VIF |
| 1 | (Constant) |  |  |
|  | Dialysis access type | ,856 | 1,168 |
|  | Diuresis(ml/24h) | ,886 | 1,129 |
|  | Albumin (g/l) | ,776 | 1,288 |
|  | Transferrin Saturation | ,877 | 1,140 |
|  | C-Reactive Protein (mg/l) | ,818 | 1,222 |
|  | Cardiovascular Disease | ,953 | 1,049 |
|  | Hospitalisation (12 months) | ,950 | 1,053 |
|  | Analgesics | ,901 | 1,110 |
|  | | | |

**Supplement Table 3:** **Self-assessment of frailty status (Frailty = VAS 3-5) by VAS compared to Fried**

|  | **non-frail by Fried** | **frail by Fried** | **total** |
| --- | --- | --- | --- |
| **VAS non-frail, n** | 37 | 3 | 40 |
| **VAS frail, n** | 33 | 49 | 82 |
| **total** | 70 | 52 | 122 |

**Supplement Figure 1: Distribution of VAS-Scores**


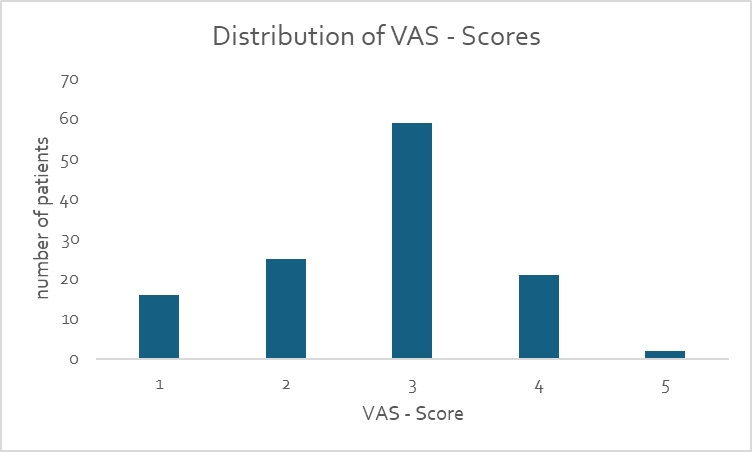

Supplement: Supplementary file 1 — Supplementary Material 1 [file 12882_2026_5218_MOESM1_ESM.docx]
